# Efficiency evaluation and redundancy analysis on low carbon economic development in Guangdong Province

**DOI:** 10.1371/journal.pone.0294112

**Published:** 2023-11-28

**Authors:** Shuang Wu, Xiangyu Yuan, Jiangwei Ni

**Affiliations:** 1 School of Economics and Management, Guangzhou Institute of Science and Technology, Guangdong, China; 2 Business Management Department, Guangdong Energy Group Co., Ltd., Guangdong, China; Jinan University, CHINA

## Abstract

Since 2020, China has been dedicated to the goal of "carbon peak and carbon neutrality" in the international community, demonstrating its commitment to energy conservation and emissions control. In 2021, in response to the guidance of the State Council, all provinces in China included the creation of a low-carbon economic system as one of their key development goals. This paper aims at investigating the low-carbon economic development efficiency and the redundancy of inefficient areas of Guangdong Province by using the three-stage DEA-Malmquist index model. Panel data of 21 cities from 2011 to 2020 in Guangdong Province were selected. Results revealed that the low-carbon economic development efficiency in the whole province rises, but the growth rate has slowed down in the past three years. Pearl River Delta area is better-developed in low-carbon economy than others. It is recommended to improve the efficiency of scale, make use of environmental advantages and develop energy-saving and emission reduction technologies.

## 1 Introduction

In the industrialization of developed countries in Europe and the United States, the concept of green economy began to sprout as early as 1972. Environmental concerns were the main focus of the Stockholm United Nations Conference on the Human Environment. However, for a long time, people still held a stereotype of "pollution first, treatment later" until they gradually start to realize that the environmental damage brought by the development of economy is irreversible. Sustainable Development Theory was a famous and profound economic theory that advocates not only the achievement of simple rapid GDP growth, but the sustainable economic and social development achieved under the premise of protecting the environment. Under such advocation, the adoption of the Kyoto Protocol in 2005 and the Copenhagen Conference on Climate Change in 2009 proved that people’s awareness of environmental protection has been aroused and deepened. The idea of green economy was widespread and was accepted by various industries in various countries, and the concept of low-carbon economy also came into being. Low-carbon economy refers to a form of economic development under the guidance of the Sustainable Development Theory, through technological innovation, industrial transformation, new energy development etc., to try best to not only reduce the consumption of coal, oil and other high-carbon energy resources, but also to reduce greenhouse gas emissions to finally balance the economic development and environment protection [1]. China put forward the goal of "carbon peak and carbon neutrality" on September 22, 2020. Up to now, it has been dedicated to the goal of "carbon peak and carbon neutrality" in the international community, demonstrating its commitment to energy conservation and emissions control. However, due to its economic and energy structure, there is still a long way to go before China can reach the goal [2].

As a province with highest GDP and rapid industrial development in China, Guangdong Province continues to take the lead in realizing the national goal of “carbon peak by 2030 and carbon neutrality by 2060". Though difficult, the province has been trying to develop its economy and protect its environment at the same time. The fact is that the proportion of fossil energy in Guangdong Province is relatively low. and its energy structure has been comparatively optimized, making it more challenging to make some changes in the future. Under such circumstances, the province will soon be under more pressure to reduce emissions and conserve energy. Therefore, it is of significance to study on the development efficiency of low-carbon economy in Guangdong Province and to further analyze the redundancy of its prefecture-level cities. This will help the province contribute to the implementation of the State Council’s 14^th^ and 15th Plan for energy conservation and emission reduction. Also, the result of the paper can provide reference value for the government to formulate corresponding emission reduction policies, which is of practical significance in the future.

## 2 Literature review

In western nations, research is being conducted on a variety of topics, including energy technology, low carbon policy, and carbon emissions, to establish a low carbon economy and increase its efficiency. Most of these studies were carried out around the UK’s Energy White Paper [3]. The UK was chosen by Dagoumas & Barker as the research subject, and the macroeconomic E3MG model was utilized to analyze various carbon emission reduction strategies [4]. According to Nakata T et al., the primary source of man-made greenhouse gases is the burning of fossil fuels, hence low-carbon energy technology should be incorporated into the restructuring of the energy sector to realize the low-carbon economy [5]. Zhu L et al. proposed five strategies for China to transform into low-carbon economy from the macro level. However, there are few evaluations on the efficiency of low-carbon economy [6]. Most efficiency assessments are conducted in a narrow segment of an industry. For instance, Lucio et al. used the SBM-DEA model to compare and analyze the low-carbon operation efficiency of numerous dairy facilities using 10 dairy plants in Italy as an example [7].

Among numerous studies on the efficiency evaluation of low-carbon economies in China, majority of researchers use Data Envelopment Analysis (DEA). Wu and Liu, Wang, Su and Wang both adopted DEA model to evaluate the low-carbon economy development in Henan and Chongqing Province respectively [8, 9]. However, DEA model cannot eliminate the environment effects and the statistical noise, which made the results hardly accurate. Therefore, a three-stage DEA model were proposed by Fred [10]. Zheng and Huang and some other researchers have used this model to evaluate the efficiency of regional low-carbon economy in China and confirmed that such model can better evaluate the effectiveness of methods for DMU models [11–15]. This was supported and explained by Chen, Yi and Liu, who said that in China, regional economic development is not balanced, the structure of energy consumption varies greatly, so it is necessary to consider the environmental effects as well as statistical noise so as to more accurately assess the carbon emissions efficiency [12]. They also proposed the combination of SBM and three stage DEA models for that SBM models are widely used in the evaluation of energy environmental efficiency [12, 14, 16–19]. Moreover, according to Yu and Yuan, the traditional DEA model that used by many researchers can only process cross-section data and cannot make dynamic comparison, so it is difficult to show the dynamic change of the efficiency of low-carbon economy [20]. Hence, a Malmquist index was added to evaluate the dynamic changes of carbon emission efficiency. This was also acknowledged and supported by Xie, Qing and Shen and Li and Zhu in their studies [19, 21]. In considering the imbalanced economic development and resources in different cities in Guangdong Province and in order to evaluate a dynamic change of low-carbon economy development efficiency, a three-stage DEA model with Malmquist index was adopted in this study.

Despite the fact that the model used is fairly constant, there are some discrepancies in the indicators and study objects chosen, which affects how conclusions are reached. The majority of academics believe Beijing and Shanghai to be the two places with the highest levels of low-carbon economic efficiency, while other provinces illustrate varying levels of performance depending on the indicators and data used in each year. For example, Xu stated that besides Beijing and Shanghai, Jiangxi, Hunan, Hainan, Chongqing and Xizang also attained the highest efficiency of low-carbon development [22] while Li and Yue concluded that Jiangsu, Guangdong and Qinghai took the lead in the efficiency of green economy in China without considering Jiangxi, Hunan and Hainan) [23]. Liu, Tu and Cheng put forward that after 2011, Tianjin also took the lead in low-carbon economic development [13].

The choice of specific provinces for the research project has greater reference value because of the differences in their policies, resources, environments, and economic structures. Zhang calculated the low-carbon economic operation efficiency of six central provinces and came to the conclusion that Hunan, Henan, Jiangxi, Shanxi, Hubei, and Anhui had the highest to lowest efficiencies [24]. Zhou, Jiang and Li collected panel data for 11 provinces and cities and concluded that there was a wide variation in the efficiency of the green economy there [25]. Yang and Wang calculated the development efficiency of low-carbon economy in Yunnan Province and discovered that redundancy, which is the waste of resources or technology, occurred in some years [26]. Zhang and Dian analyzed the efficiency of low-carbon economy in Guangdong Province and concluded that the efficiency of low-carbon economy in the Pearl River Delta area is lower than that in the east and west of Guangdong [27]. In order to increase the general effectiveness of its low-carbon economy, this study takes Guangdong Provinces the research object.

To sum up, western researchers cover a wide range in terms of low-carbon economy, but there are few researches on efficiency evaluation, among which empirical studies can hardly be seen. Though Chinese researchers carried out relatively more Efficiency evaluation studies, the research results are difficult to be representative and have limited reference value due to differences in research objects, too few input and output indicators, too few or too old data. Therefore, based on previous studies, this paper carries out an empirical analysis using Guangdong Province as the research object, and constructs a three-stage DEA-Malmquist index model to calculate both the static efficiency and dynamic efficiency changes of input-output of low-carbon economic development in prefecture-level cities of Guangdong Province. This paper can make a more comprehensive analysis on Guangdong Province’s low-carbon economic efficiency, and provide reference value to some extent.

## 3 Research method

Data Envelopment Analysis Approach (DEA) was a research methodology that widely used in many industries like economy, international business, manufacturing etc. to help evaluate the relative effectiveness among same decision-making departments. This method was initially proposed by Charnes & Rhodes and was especially efficient in evaluating those with multiple inputs and outputs [28]. Three stage DEA model was further proposed by Fried on such basis for that the original DEA model cannot eliminate the effects of environmental factors nor can it eliminate the statistical noise [10]. Therefore, the study adopted the three-stage DEA model as the research methodology which can more accurately reflect the change in efficiency.

### 3.1 Stage 1: DEA-BCC model

According to the change of returns to scale, the traditional DEA model can be classified into two categories: CCR model (unchanged returns to scale) and BCC model (variable returns to scale). According to the actual scenario, the return to scale influences the comprehensive technical efficiency of DMU, thus the DEA-BCC model is chosen in the assessment study on the development efficiency of low-carbon economy in Guangdong Province. This model implies that there are n DMUs, and that there are m input indications and t output indicators for any DMUk (k = 1, 2… n). The model can be expressed as:

$$\min\theta -\varepsilon (\sum_{i=1}^{m}S_{i}^{-}+\sum_{r=1}^{t}S_{r}^{+})$$ (1)

Constraint conditions are:

$$s.t.\{\begin{array}{l} \sum_{k=1}^{n}\lambda_{k}x_{ik}+S_{i}^{-}=\theta x_{ik0},i=1,2,....,m\\ \sum_{k=1}^{n}\lambda_{k}y_{rk}-S_{r}^{-}=y_{rk0},r=1,2,....,t\\ \lambda_{k}\geq 0,k=1,2,...,n\\ S_{i}^{-}\geq 0,S_{r}^{+}\geq 0 \end{array}$$ (2)

We denote efficiency as *Θ*, input as *x*_*ik*_, output as *y*_*rk*_, slack variable as *S+ r*, surplus variable as *S- i*, coefficient as *λ*_*k*_, Non Archimedean infinitesimal quantity as *ε*. In the above model, the optimal solution of parameters to be estimated has the following meanings:

1. when *θ* = 1 and *S+ r* = *S- i* = 0, the decision unit DMUk is DEA efficient, and it is both technology efficient and scale efficient;
2. when *θ* = 1 and *S+ r*, *S- i* if at least one decision unit DMUk is not 0, the effectiveness of DEA is weak;
3. when *θ*<1, decision unit DMUk is not DEA efficient.

### 3.2 Stage 2: SFA regression model

Fred proposed SFA regression model to be effective in separating both the environmental effects and the statistical noise [10]. In stage 2, SFA regression model is used to eliminate the environmental effects and statistical noise in the first stage to make the results more statistical effective. Environmental factors will be taken as explanatory variable, and the slack variable of input indicators in Stage 1 as the explained variable. The SFA model is built to analyze the redundancy of decision-making unit. The formula is as follows:

$$S_{ik}=f(Z_{k};\beta_{i})+\nu_{ik}+\mu_{ik}$$ (3)

*S*_*ik*_ represents the redundancy of the i-th input index in the k-th decision unit in the first-stage model, *Z*_*k*_ is environment variable, *β*_*i*_ is regression parameters to be estimated, *ν*_*ik*_+*μ*_*ik*_ is mixed error term, *ν*_*ik*_ is random disturbance, and *μ*_*ik*_ refers to management inefficiency.

The initial input value was modified in accordance with the regression parameters of the explanatory variables obtained from the regression model in order to remove the influence of environmental factors and random factors on the measurement efficiency. The formula is as below:

$$X_{ik}^{A}=X_{ik}+\{\max[f(Z_{k};{\hat{\beta }}_{i})]-f(Z_{k};{\hat{\beta }}_{i})\}+[\max(\nu_{ik})-\nu_{ik}]$$ (4)

*X*_*ik*_ is the original value of the i-th input index of the k-th decision unit, *XA ik* is the adjusted value.

### 3.3 Stage 3: Adjusting the original value and re-calculation

After eliminating the environmental effects and the statistical noise, the DEA-BCC model ran again to calculate the adjusted input and output variables in stage 2. Then the adjusted efficiency value was acquired and compared to the first stage’s outcome. The adjusted index value is also used to generate the Malmquist index.

### 3.4 Malmquist index

Malmquist index was first proposed by Malmquist in 1953 [29]. It is an analytical method used to dynamically evaluate the efficiency of objects and measure the total factor productivity of decision-making unit. This was used to measure changes in productivity in the first place [30]. With the advantage of inter-period comparison, it can overcome the shortcoming of traditional DEA and process cross section data [31]. Malmquist index formula from the t period to the t+1 period can be expressed as follows:

$$M_{0}^{t+1}=\sqrt{\frac{D^{t}(x_{0}^{t+1},y_{0}^{t+1})}{D^{t}(x_{0}^{t},y_{0}^{t})}\times \frac{D^{t+1}(x_{0}^{t+1},y_{0}^{t+1})}{D^{t+1}(x_{0}^{t},y_{0}^{t})}}$$ (5)

Assuming constant returns to scale, total factor productivity (TFP) can be decomposed into the product of technical efficiency change (effech) and technological progress (tech),

$$\begin{array}{l} M_{0}^{t+1}=\frac{D^{t}(x_{0}^{t+1},y_{0}^{t+1})}{D^{t}(x_{0}^{t},y_{0}^{t})}\sqrt{\frac{D^{t}(x_{0}^{t+1},y_{0}^{t+1})}{D^{t+1}(x_{0}^{t+1},y_{0}^{t+1})}\times \frac{D^{t}(x_{0}^{t},y_{0}^{t})}{D^{t+1}(x_{0}^{t},y_{0}^{t})}}\\ =TFP=effech\times tech \end{array}$$ (6)

Assuming that returns to scale are variable, technical efficiency change (effech) can be decomposed into the product of pure technical efficiency (pech) and scale efficiency (sech) product,

$$\begin{array}{l} effch=pech\times \mathrm{sech}\\ TFP=effch\times tech=pech\times \mathrm{sech}\times tech \end{array}$$ (7)

The parameters of the above model have the following meanings:

When TFP>1, it means that the productivity of the decision-making unit has increased from the t period to the t+1 period. When TFP = 1, the productivity level remains unchanged, and vice versa;

effech reflects the change of technical efficiency of DMU, and tech reflects the technical progress or regression of DMU;

pech reflects the change of pure technical efficiency of decision-making unit, and sech reflects the change of scale efficiency. When sech>1, it means that the decision-making unit is closer to the fixed scale return from t to t+1, and vice versa.

## 4 Data source and index selection

### 4.1 Data source

The Guangdong Statistical Yearbook and the Prefecture-Level City Statistical Yearbooks are the main sources of the data. The main selection includes panel data for 21 prefecture-level cities in Guangdong Province from 2011 to 2020. The perpetual inventory method is used to determine the stock data for fixed assets [32]. Data on carbon dioxide emissions are sourced from the CEADS (China Carbon Accounting Database). The Intergovernmental Panel on Climate Change (IPCC) released the IPCC Guidelines for National Greenhouse Gas Inventories, 2006, 2019 Revised Edition in order to account for any years where carbon emission data may be absent [33–35].

### 4.2 Index selection

The following indicators were chosen mostly based on the results of earlier studies by Xu [22], Li and Yue [23], and Zhang and Dian [27], as well as the actual circumstances:

(1) Input indicators: The number of employees at the end of the year, the stock of fixed assets and the total energy consumption were used to demonstrate the three basic production factors of labor, capital, and energy respectively.

(2) Output indicators: GDP is utilized to reflect economic aggregate, and total societal energy consumption was also chosen. The two indicators of the predicted output are those two. Direct electricity consumption results in energy use without carbon emissions. When the primary energy consumption is given, the total energy consumption of the entire society indicates the efficiency of energy use without carbon emissions and can be used to demonstrate how much a society reduces carbon dioxide emissions during energy consumption. Therefore, output index of the effectiveness of the development of the low-carbon economy is therefore the total energy consumption of the entire society. Emissions of carbon dioxide are utilized as a sign of unexpected results.

(3) Environmental factor: Due to the influence of external environment on the efficiency of low-carbon economy, environmental variables are selected from five aspects: urbanization level, industrial structure, technological innovation, government regulation and economic openness. The specific definition is shown as follow:

1. Urbanization level: the proportion of urban population in the permanent population of this city;
  Industrial structure: the proportion of output value of secondary industry in total output value;
2. Technological innovation: the proportion of R&D input in the total output of industrial enterprises above designated size;
3. Government regulation: the proportion of local general public budget expenditure in GDP;
4. Economic openness: the proportion of total imports and exports in GDP.

The specific description of each indicator is shown below in Table 1.

**Table 1:** Input-output indicators for low-carbon economy.

Indicators	Variable
Input Indicators	Number of Employees at Year-end
	Stock of Fixed Assets
	Total Energy Consumption
Output Indicators	GDP
	Total Electricity Consumption
	Carbon Dioxide Emissions
Environmental Factors	Urbanization Level
	Industrial Structure
	Technological Innovation
	Governmental Regulation
	Economic Openness

## 5 Empirical analysis

After analyzing the development efficiency of low-carbon economy in Guangdong Province, the involved prefecture-level cities are divided according to the statistical areas classified by Guangdong Provincial Bureau of Statistics. Guangdong Province is hence divided into Pearl River Delta, East Guangdong, West Guangdong and North Guangdong. The details are displayed in Table 2.

**Table 2:** 21 prefecture-level cities and geographical divisions of Guangdong Province.

Area	Prefecture-level Cities
Pearl River Delta	Guangzhou, Shenzhen, Zhuhai, Foshan, Huizhou, Dongguan, Zhongshan, Jiangmen, Zhaoqing
East Guangdong	Shantou, Shanwei, Chaozhou, Jieyang
West Guangdong	Yangjiang, Zhanjiang, Maoming
North Guangdong	Shaoguan, Heyuan, Meizhou, Qingyuan, Yunfu

### 5.1 First stage: Empirical analysis results of DEA-BCC model

(1) Comprehensive technical efficiency Value. Under the condition that returns to scale varies, the DEA-BCC model is used to calculate the panel data of 21 prefecture-level cities in Guangdong Province from 2011 to 2020, and the results are shown in Table 3.

**Table 3:** Comprehensive technical efficiency of Guangdong Province.

City/Area	2011	2012	2013	2014	2015	2016	2017	2018	2019	2020
Guangzhou	1.00	1.00	1.00	1.00	1.00	1.00	1.00	0.98	1.00	1.00
Shenzhen	1.00	1.00	1.00	1.00	1.00	1.00	1.00	1.00	1.00	1.00
Zhuhai	0.95	0.92	0.92	0.94	0.96	0.96	1.00	1.00	1.00	0.95
Shantou	1.00	0.98	0.95	0.91	0.85	0.81	0.75	0.71	0.67	0.67
Foshan	1.00	1.00	1.00	1.00	1.00	1.00	1.00	1.00	1.00	1.00
Shaoguan	0.67	0.67	0.74	0.76	0.71	0.69	0.71	0.79	0.82	0.91
Heyuan	0.68	0.66	0.75	0.76	0.73	0.72	0.68	0.65	0.67	0.67
Meizhou	0.67	0.65	0.64	0.63	0.60	0.58	0.56	0.53	0.52	0.53
Huizhou	0.78	0.79	0.81	0.84	0.84	0.88	0.92	1.00	1.00	1.00
Shanwei	0.80	0.78	0.76	0.72	0.64	0.64	0.60	0.60	0.59	0.56
Dongguan	1.00	1.00	1.00	1.00	1.00	1.00	1.00	1.00	1.00	1.00
Zhongshan	1.00	1.00	1.00	1.00	1.00	1.00	1.00	1.00	1.00	1.00
Jiangmen	0.78	0.79	0.82	0.86	0.82	0.81	0.80	0.81	0.82	0.86
Yangjiang	0.54	0.58	0.61	0.65	0.68	0.71	0.72	0.76	0.91	0.95
Zhanjiang	0.76	0.70	0.63	0.60	0.57	0.52	0.51	0.51	0.51	0.52
Maoming	1.00	1.00	0.98	0.82	0.68	0.58	0.54	0.54	0.55	0.57
Zhaoqing	1.00	0.88	0.79	0.77	0.67	0.68	0.66	0.63	0.62	0.59
Qingyuan	0.65	0.68	0.75	0.80	0.80	0.86	0.75	0.82	0.85	0.92
Chaozhou	0.70	0.70	0.70	0.68	0.67	0.67	0.66	0.70	0.70	0.73
Jieyang	0.80	0.81	0.79	0.79	0.69	0.68	0.59	0.64	0.60	0.63
Yunfu	0.41	0.40	0.41	0.41	0.40	0.41	0.42	0.44	0.46	0.50
Pearl River Delta	0.95	0.93	0.93	0.93	0.92	0.93	0.93	0.93	0.94	0.93
East Guangdong	0.82	0.82	0.80	0.77	0.71	0.70	0.65	0.66	0.64	0.65
West Guangdong	0.77	0.76	0.74	0.69	0.64	0.60	0.59	0.60	0.66	0.68
North Guangdong	0.62	0.61	0.66	0.67	0.65	0.65	0.62	0.65	0.66	0.70
Mean of the province	0.82	0.81	0.81	0.81	0.78	0.77	0.75	0.77	0.78	0.79

According to the results, first, the comprehensive efficiency value of low-carbon economic development in Shenzhen, Foshan, Dongguan and Zhongshan were DEA effective. With the exception of 2018, Guangzhou was DEA-compliant. This shows that the development of a low-carbon economy is a priority for the aforementioned five cities in the entire province. DEA became effective in Zhuhai between 2017 and 2019 and in Huizhou between 2018 and 2020. Shantou and Zhaoqing only became DEA effective in 2011, while Maoming became DEA between 2011 and 2012. Yunfu has the lowest efficiency value. Its complete efficiency value on average is 0.426, with Meizhou coming in second at 0.591. Second, nine prefecture-level cities- Guangzhou, Shenzhen, Zhuhai, Shantou, Foshan, Huizhou, Dongguan, Zhongshan, and Jiangmen- have comprehensive technical efficiency values that are higher than the provincial average. 12 cities are lower than the provincial average value, among which 3 belong to East Guangdong area. The value of all the cities of West Guangdong and North Guangdong area are lower than the average value of the province. 12 cities are lower than the provincial average value. Therefore, Pearl River Delta is superior to East Guangdong, West Guangdong, and North Guangdong in terms of low-carbon economic development. Thirdly, until 2017, Guangdong Province’s average comprehensive efficiency of low-carbon economic development declined; from 2017 to 2020, it rose.

(2) The value of pure technical efficiency. First, nine cities, including Guangzhou, Shenzhen, Zhuhai, Foshan, Heyuan, Shanwei, Dongguan, Zhongshan, and Chaozhou, achieved pure technical efficiency values of 1, while Yangjiang and Yunfu could achieve this value for most of the years. Because Yunfu’s comprehensive technical efficiency is the lowest in the province and indicates that it is primarily dependent on economies of scale, it has undergone the most transformation of the other 5 cities. Jiangmen, Zhanjiang, and Jieyang all have values that are consistently lower than the province’s mean (0.95); in particular, Zhanjiang’s rating fell between 0.6 and 0.65 between 2016 and 2020, making it the lowest in the province with an average pure technical efficiency of 0.745. Second, there are 12 cities, including Guangzhou, Shenzhen, Zhuhai, Foshan, Shaoguan, Heyuan, Shanwei, Dongguan, Zhongshan, Yangjiang, Chaozhou, and Yunfu, whose pure technical efficiency value is higher than the provincial average level. Of these, 6 are in the Pearl River Delta area and 2 are in the East China Plain. As a result, the Pearl River Delta region’s technical efficiency is higher than that of the North and East Guangdong regions. Additionally, the value is significantly lower than the provincial average in the West Guangdong region. Thirdly, the province’s average pure technical efficiency from 2011 to 2014 of 0.97 remained constant. It begins to exhibit a downward trend in 2015 and remained at 0.93 from 2017 to 2019 before marginally increasing to 0.94 in 2020, but it has not yet reached the level of 2011.

(3) Scale efficiency and returns to scale. Firstly, the five cities with the highest scale efficiency are Guangzhou, Shenzhen, Foshan, Dongguan, and Zhongshan, whose value remains constant at one every ten years. As a result, we can see that these five cities’ scale of development is generally appropriate. Therefore, the development of scale in these 5 cities are relatively reasonable. The value of scale efficiency of Zhuhai is 1 in 2018–2019 while Huizhou reached 1 in 2018–2020, Maoming reached 1 in 2011–2012, Shantou and Zhaoqing reached 1 in 2021. The scale efficiency is ineffective for other cities. Secondly, in Guangdong Province, the scale efficiency of low-carbon economic development remained constant at 0.81 to 0.85. East Guangdong, West Guangdong, and North Guangdong have scale efficiency values that are below the provincial mean, which is consistent with the performance of the comprehensive efficiency result, while the Pearl River Delta area has a value that is higher than the average regional level and above average at the provincial level. Thirdly, from the perspective of returns to scale, all the cities remained the same or grew from the perspective of returns to scale. Therefore, it is crucial to increase scale input and scale efficiency for cities with scale efficiency values below 1.

### 5.2 Stage 2: Empirical analysis results of SFA regression model influenced by external environmental factors

Since each city has a unique external environment, software Frontier 4.1 is used to run the SFA regression model to perform regression analysis using the maximum likelihood method on the cross-section data for each year from 2011 to 2020. This further excludes the influence of environmental factors on the results. Five environmental factors were employed as explanatory variables while the slack value of the input variable calculated in the first stage is taken as the explained variable. In addition, carbon dioxide emissions are considered as undesirable outputs. If the slack value of the input variable is positively correlated with the environmental variable, this suggests that an increase in the environmental variable will reduce the effectiveness of the creation of a low-carbon economy.; conversely, if they are negatively correlated, it means that the environmental variable’s increase will increase the efficiency of the development of the low-carbon economy.

The results of SFA regression analysis are as follows. Due to the limited space here, only data of 2019 and 2020 are presented in this paper, as shown in Tables 4 and 5.

**Table 4:** Regression results of SFA in 2019.

	Slack Value of Fixed Assets Stock		Slack Value of the Number of Employed Persons at The End of The Year		Slack Value of Total Energy Consumption		Slack Value of Carbon Dioxide Emission	
	Coefficient	T-value	Coefficient	T-value	Coefficient	T-value	Coefficient	T-value
Constant	181.14	181.12***	23.06	20.35***	49.04	49.68***	-3.38	-2.373**
Urbanization	-12.16	-14.60***	-0.24	-6.02***	-0.27	-0.75	-0.03	-4.602***
Industrial structure	8.03	6.45***	-0.32	-4.89***	-1.00	-5.29***	0.07	3.381***
Science and Technology Input	26.38	26.48***	0.54	1.61	-0.49	-0.55	0.22	4.230***
Governmental Regulation	-8.20	-8.72***	-0.26	-1.32	-0.31	-2.53**	0.08	3.295***
Economic Openness	-0.35	-0.29	0.02	0.29	0.28	8.33***	-0.03	-2.802**
x2	228598	228598***	291	290***	3192	3192	28.12	8.552***
gamma	0.999	2.04E+05***	0.999	8.82E+04***	0.999	1.87E+06***	0.999	2.59E+06***
Log Likelihood	-145.45		-70.86		-94.92		-50.21	
LR	8.67		17.54		20.03		19.48	

**Table 5:** Regression results of SFA in 2020.

	Slack Value of Fixed Assets Stock		Slack Value of the Number of Employed Persons at The End of The Year		Slack Value of Total Energy Consumption		Slack Value of Carbon Dioxide Emission	
	Coefficient	T-value	Coefficient	T-value	Coefficient	T-value	Coefficient	T-value
Constant	-117.20	-117.19***	33.67	33.62***	145.47	145.49***	-6.20	-4.22***
Urbanization	-11.38	-8.18***	-0.32	-1.30	-0.58	-1.00	0.04	0.51
Industrial structure	11.86	3.62***	-0.44	-1.74	-2.54	-2.76***	0.04	0.96
Science and Technology Input	44.82	39.57***	0.34	0.34	-1.81	-1.83*	0.12	0.58
Governmental Regulation	-4.34	-1.64***	-0.41	-1.34	-1.92	-1.97*	0.10	0.79
Economic Openness	-4.40	-9.36***	0.09	0.75	0.58	0.88	-0.05	-1.319
x2	303054	303054***	340	340***	11160	111600***	48.16	14.282***
gamma	0.999	1.10E+04***	0.999	2.75E+05***	0.999	6.31E+04***	0.999	1.27E+06***
Log Likelihood	-147.54		-73.16		-108.90		-51.69	
LR	10.08		16.20		18.02		18.48	

Note: ***, **, * indicates that it is significant respectively at 1%, 5% and 10%

The effectiveness of SFA regression is demonstrated by the fact that all LR unilateral error amounts pass the LR test at the significance level of 1%, as indicated in the table above. Since the Gamma value is so near to 1, it is imperative to rule out environmental influences because they contribute significantly to the mixed error term. The findings demonstrate that many environmental conditions affect each input variable’s slack values in a variety of ways. From 2011 through 2020, many statistically significant associations were also given.

Urbanization levels strongly and negatively relate to employment, total energy consumption, fixed asset stock slack value, and carbon dioxide emissions at the 1% level in several years. This suggests that as urbanization increases, fixed asset, energy, and labor utilization rates rise, carbon dioxide emissions decrease and low-carbon economic development becomes more effective. At the 1% level, there is a substantial negative association between the industrial structure and carbon dioxide emissions and a significant positive correlation between the industrial structure and the stock of slack fixed assets. It shows that the secondary industry is dominated by the industry. Most of the actors in the secondary industries are capital-intensive. The stock of fixed assets will increase along with the proportion of secondary industries. The slack value of fixed asset stock, science and technology investment, and carbon dioxide emissions all significantly positively correlate at the 1% level. The stock of fixed assets and carbon dioxide emissions will both rise in line with an increase in the share of scientific research investment made by industrial businesses over the designated size, for this reason. Government regulations have different effects on various variables in different years, and they are negatively correlated with the stock of slack fixed assets. This suggests that the government intends to use policies to direct the redundancy of fixed assets in order to increase the efficiency with which the low-carbon economy develops. The degree of economic openness is positively or negatively correlated with different variables at different significant levels in different years, which is not representative.

To sum up, different environmental factors have a significant impact on each input index in different years. Therefore, this difference will affect the efficiency evaluation of low-carbon economic development in different areas under different external environments. It is thus more representative to calculate and evaluate the original input index after adjusting for the influence of environmental factors and random errors.

### 5.3 The comparative analysis on the result of Stage 1 and Stage 3

**Fig 1:**
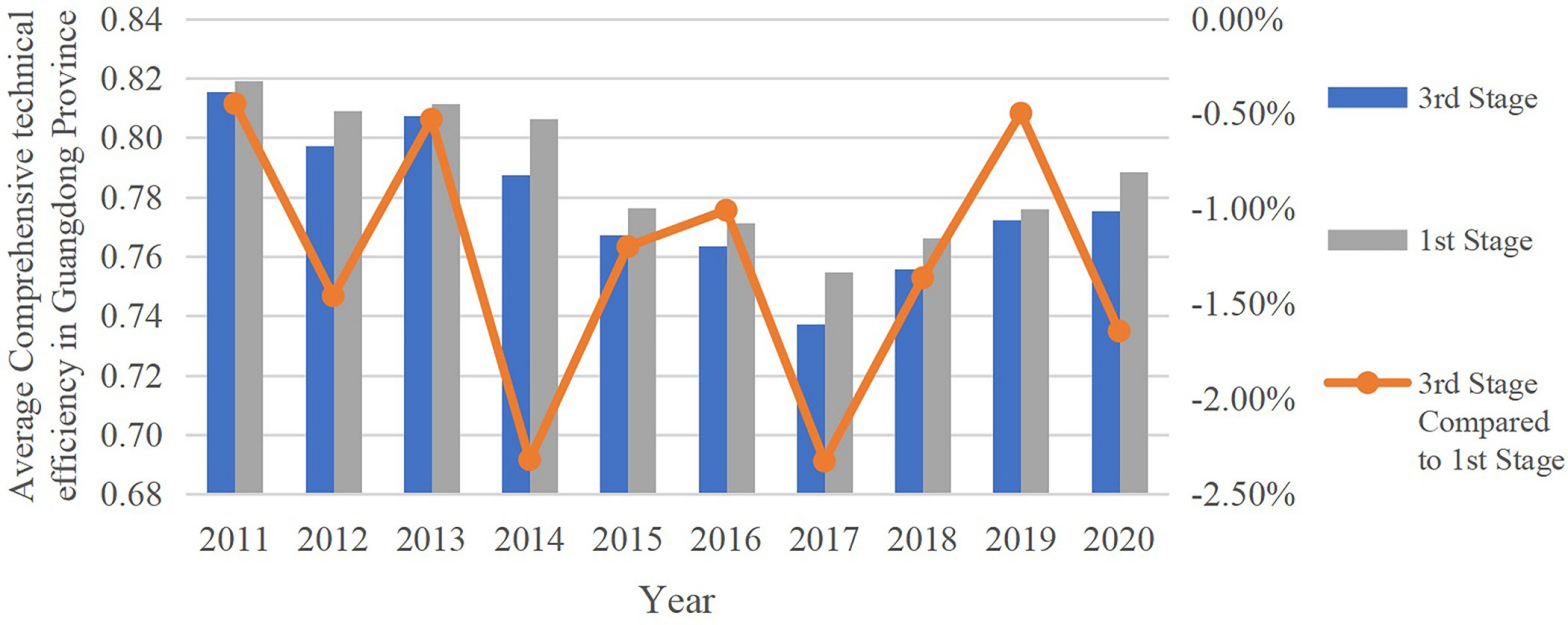
Comparison of provincial average comprehensive technical efficiency of 3^rd^ and 1^st^ stage.

The DEA model is used to calculate the input index after excluding the environmental factors. The results obtained and the changes between comprehensive technical efficiency of Stage 1 are shown in Table 6 and Fig 1.

**Table 6:** Change rate of comprehensive technical efficiency of the 3^rd^ compared with that of the 1^st^ stage for cities and areas.

City/Area	2011	2012	2013	2014	2015	2016	2017	2018	2019	2020
Guangzhou	0.00%	0.00%	0.00%	0.00%	0.00%	0.00%	0.00%	1.47%	0.00%	0.00%
Shenzhen	0.00%	0.00%	0.00%	0.00%	0.00%	0.00%	0.00%	0.00%	0.00%	0.00%
Zhuhai	0.00%	0.00%	0.00%	-0.46%	-5.23%	0.00%	0.00%	-0.53%	0.00%	-3.27%
Shantou	0.00%	-2.46%	-1.50%	-5.56%	-1.63%	-2.59%	-6.53%	-2.25%	-0.97%	-1.96%
Foshan	0.00%	0.00%	0.00%	0.00%	0.00%	0.00%	0.00%	0.00%	0.00%	0.00%
Shaoguan	0.00%	0.00%	0.00%	-1.62%	-2.13%	0.00%	-0.39%	-2.93%	1.38%	-3.29%
Heyuan	0.29%	-3.47%	-0.59%	-7.30%	-0.96%	-1.63%	-9.54%	-3.29%	-4.71%	-4.32%
Meizhou	-0.55%	-0.98%	-0.34%	-7.76%	-2.08%	-3.78%	-12.42%	-4.80%	-3.40%	-5.44%
Huizhou	-0.04%	0.00%	0.00%	1.19%	1.24%	0.00%	0.23%	0.21%	0.00%	0.00%
Shanwei	-5.90%	-14.56%	-8.45%	-23.31%	-7.19%	-11.67%	-22.56%	-11.33%	-1.40%	-5.67%
Dongguan	0.00%	0.00%	0.00%	0.00%	0.00%	0.00%	0.00%	0.00%	0.00%	0.00%
Zhongshan	0.00%	0.00%	0.00%	0.00%	0.00%	0.00%	0.00%	0.00%	0.00%	0.00%
Jiangmen	-0.06%	0.00%	0.00%	-0.92%	0.25%	-0.17%	0.12%	-0.38%	3.05%	2.27%
Yangjiang	-0.32%	-3.07%	-0.05%	-2.69%	-1.44%	0.00%	-0.35%	-1.58%	-1.87%	-4.01%
Zhanjiang	-0.18%	-0.67%	0.00%	-2.99%	-1.14%	-2.04%	-3.48%	-0.56%	0.38%	0.88%
Maoming	0.00%	0.00%	0.00%	0.17%	0.00%	0.00%	-0.06%	-0.47%	0.72%	-1.70%
Zhaoqing	0.00%	-4.59%	-0.75%	-1.39%	0.16%	-1.55%	-3.10%	-1.79%	0.82%	0.93%
Qingyuan	0.00%	0.00%	0.00%	1.38%	-0.42%	0.00%	-0.32%	-0.72%	0.18%	-1.46%
Chaozhou	-0.18%	-0.03%	0.00%	-2.09%	-6.26%	0.00%	-1.00%	-4.99%	-7.79%	-10.48%
Jieyang	-2.50%	-0.32%	0.84%	-0.05%	0.00%	-1.70%	-0.09%	-0.31%	0.88%	0.79%
Yunfu	-0.66%	-3.84%	-1.33%	-1.66%	-0.67%	0.00%	-0.24%	-0.50%	0.10%	-1.06%
Pearl River Delta	-0.01%	-0.48%	-0.07%	-0.15%	-0.44%	-0.14%	-0.21%	-0.04%	0.36%	-0.07%
East Guangdong	-2.08%	-4.29%	-2.24%	-7.54%	-3.58%	-3.82%	-7.36%	-4.56%	-2.50%	-4.49%
West Guangdong	-0.14%	-0.99%	-0.01%	-1.64%	-0.84%	-0.58%	-1.16%	-0.96%	-0.57%	-2.12%
North Guangdong	-0.14%	-1.46%	-0.37%	-3.34%	-1.25%	-1.03%	-4.50%	-2.42%	-1.08%	-3.01%
Mean of the province	-0.45%	-1.46%	-0.53%	-2.32%	-1.20%	-1.01%	-2.33%	-1.36%	-0.50%	-1.64%

#### 5.3.1 Comparative analysis on comprehensive technical efficiency in Stage 1 and 3

Firstly, the average total technical efficiency of the 21 prefecture-level cities in Guangdong Province decreased when the impact of environmental variables is taken into account, demonstrating the province’s favorable climate for the growth of low-carbon economies. Secondly, Shenzhen, Foshan, Dongguan and Zhongshan demonstrate no changes in Stage 3 compared with Stage 1. Therefore, environmental factors have no impact on the efficiency of low-carbon economic development in these four cities. From Stage 1 to Stage 3, Guangzhou’s average comprehensive technical efficiency increased by 0.14%, primarily as a result of the influence of the value in 2018. With the exception of 2018, Guangzhou attained DEA in all other years. Huizhou’s average comprehensive technological efficiency, nevertheless, improves 0.27% in Stage 1 compared with that of Stage 3. It demonstrates that environmental factors had a detrimental effect on these two cities, and if the environmental elements were not considered, the results would have been underestimated. Other prefecture-level cities’ average comprehensive efficiencies also declined, but to varied degrees; Shanwei saw the most loss, at 11.2%. The results could be inflated because environmental factors have a significant impact on these communities and are often neglected. Thirdly, all locations experience a loss in overall technical efficiency after environmental considerations are taken into account, with East Guangdong experiencing the biggest decrease (4.2%) due to Shantou, Shanwei, and Chaozhou. The rate of decline is higher in the North Guangdong area than the provincial average, at 1.87%, while it is lower in the West Guangdong and Pearl River Delta areas, at 0.89% and 0.13%, respectively. As a result, environmental factors are more beneficial in the East Guangdong region while having a relatively smaller impact on the Pearl River Delta region.

#### 5.3.2 Comparative analysis of pure technical efficiency in 3^rd^ and 1^st^ stage

**Fig 2:**
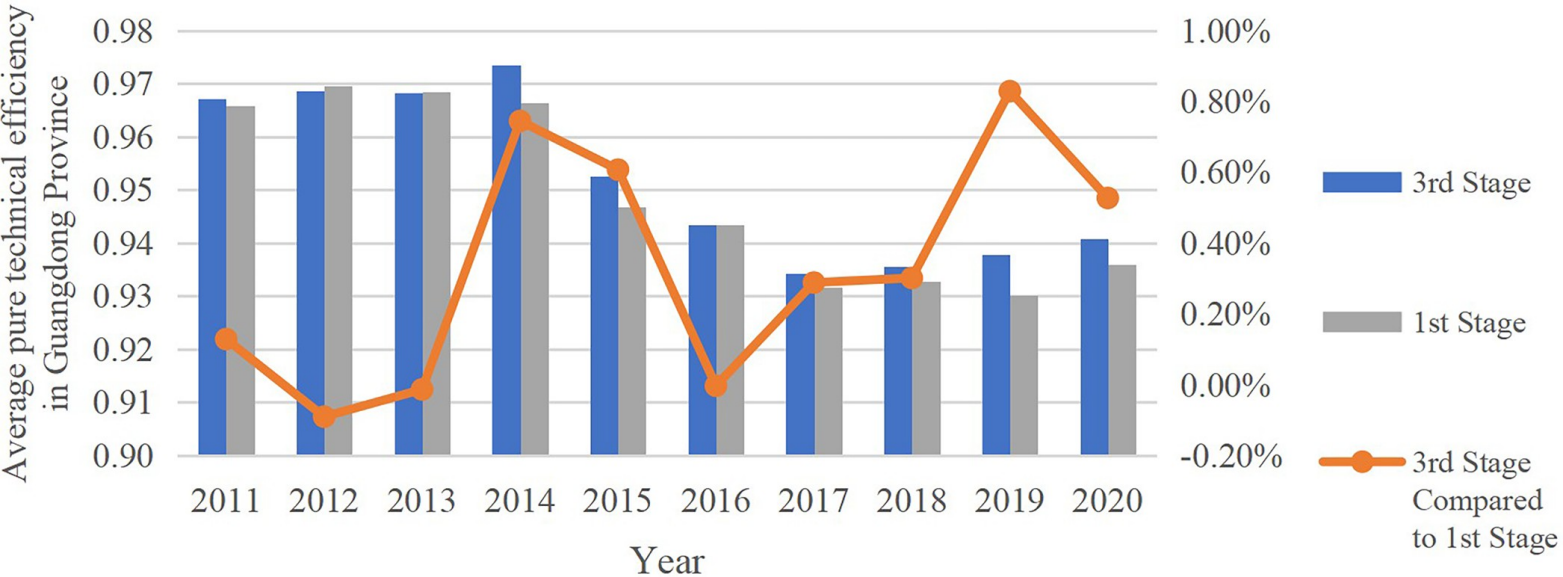
Comparison of provincial average pure technical efficiency of 3^rd^ and 1^st^ stage.

As shown in Table 7 and Fig 2, after excluding the influence of environmental factors, firstly, except 2012–2013, the average pure technical efficiency of the province in the Stage 3 decreased compared with that of Stage 1. In other years, the average pure technical efficiency of the province in Stage 3 all increased compared with that of Stage 1. Secondly, average pure technical efficiency in Guangzhou, Shenzhen, Zhuhai, Foshan, Shaoguan, Heyuan, Shanwei, Dongguan, Zhongshan and Chaozhou remain unchanged in Stage 3 when compared with that in Stage 1, which indicates that environmental factors have no influence on the pure technical efficiency of the above 10 cities. Average pure technical efficiency of Shantou and Meizhou decreased in Stage 3 at respectively 0.73% and 0.94%. Therefore, environmental factors have positive impact on the pure technical efficiency of Meizhou and Shantou to some extent. Compared with Stage 1, the average pure technical efficiency of the other 9 cities in the Stage 3 increased to different degrees, and the largest increase was Maoming at 1.62%. Environmental factors had a negative effect on them. Thirdly, Environmental factors has a more negative impact on West Guangdong area than that on Pearl River Delta, North Guangdong and East Guangdong area.

**Table 7:** Change rate of pure technical efficiency of the 3^rd^ compared with that of the 1^st^ stage for cities and areas.

City/Area	2011	2012	2013	2014	2015	2016	2017	2018	2019	2020
Guangzhou	0.00%	0.00%	0.00%	0.00%	0.00%	0.00%	0.00%	0.00%	0.00%	0.00%
Shenzhen	0.00%	0.00%	0.00%	0.00%	0.00%	0.00%	0.00%	0.00%	0.00%	0.00%
Zhuhai	0.00%	0.00%	0.00%	0.00%	0.00%	0.00%	0.00%	0.00%	0.00%	0.00%
Shantou	0.00%	0.00%	0.00%	-1.19%	-1.21%	-1.87%	-1.56%	-1.08%	-0.17%	-0.34%
Foshan	0.00%	0.00%	0.00%	0.00%	0.00%	0.00%	0.00%	0.00%	0.00%	0.00%
Shaoguan	0.00%	-0.09%	0.00%	0.80%	0.60%	-0.81%	0.03%	0.00%	1.20%	-1.72%
Heyuan	0.00%	0.00%	0.00%	0.00%	0.00%	0.00%	0.00%	0.00%	0.00%	0.00%
Meizhou	0.14%	-3.27%	-1.33%	-2.89%	0.27%	-0.30%	-1.60%	-0.79%	-0.08%	0.83%
Huizhou	0.70%	0.00%	0.00%	3.70%	3.53%	0.00%	0.00%	0.00%	0.00%	0.00%
Shanwei	0.00%	0.00%	0.00%	0.00%	0.00%	0.00%	0.00%	0.00%	0.00%	0.00%
Dongguan	0.00%	0.00%	0.00%	0.00%	0.00%	0.00%	0.00%	0.00%	0.00%	0.00%
Zhongshan	0.00%	0.00%	0.00%	0.00%	0.00%	0.00%	0.00%	0.00%	0.00%	0.00%
Jiangmen	0.67%	0.32%	0.06%	2.02%	1.67%	-0.04%	1.62%	0.49%	2.12%	2.57%
Yangjiang	0.00%	-0.12%	0.00%	1.25%	0.00%	-0.90%	0.00%	0.00%	0.00%	0.00%
Zhanjiang	0.17%	-0.31%	-0.09%	1.09%	0.42%	-0.16%	2.67%	2.73%	2.98%	4.52%
Maoming	0.00%	0.00%	0.00%	0.00%	0.00%	3.53%	3.58%	2.77%	5.10%	2.68%
Zhaoqing	0.00%	0.00%	0.40%	4.06%	3.73%	0.18%	0.81%	0.81%	2.09%	2.20%
Qingyuan	0.41%	0.00%	0.00%	5.43%	3.85%	0.00%	0.15%	1.61%	2.27%	0.65%
Chaozhou	0.00%	0.00%	0.00%	0.00%	0.00%	0.00%	0.00%	0.00%	0.00%	0.00%
Jieyang	0.86%	1.61%	0.73%	2.57%	1.05%	0.69%	1.94%	-0.47%	2.17%	2.19%
Yunfu	0.00%	0.00%	0.00%	0.00%	0.00%	0.00%	0.00%	1.46%	1.63%	0.00%
Pearl River Delta	0.13%	0.03%	0.05%	1.01%	0.89%	0.01%	0.24%	0.12%	0.41%	0.46%
East Guangdong	0.21%	0.39%	0.17%	0.29%	-0.08%	-0.29%	0.07%	-0.36%	0.45%	0.44%
West Guangdong	0.06%	-0.14%	-0.03%	0.76%	0.12%	0.80%	1.88%	1.62%	2.51%	2.05%
North Guangdong	0.10%	-0.70%	-0.26%	0.62%	0.91%	-0.22%	-0.27%	0.47%	1.01%	-0.07%
Mean of the province	0.13%	-0.09%	-0.01%	0.74%	0.61%	0.00%	0.29%	0.30%	0.83%	0.53%

#### 5.3.3 Comparative analysis of scale efficiency in 3^rd^ and 1^st^ stage

**Fig 3:**
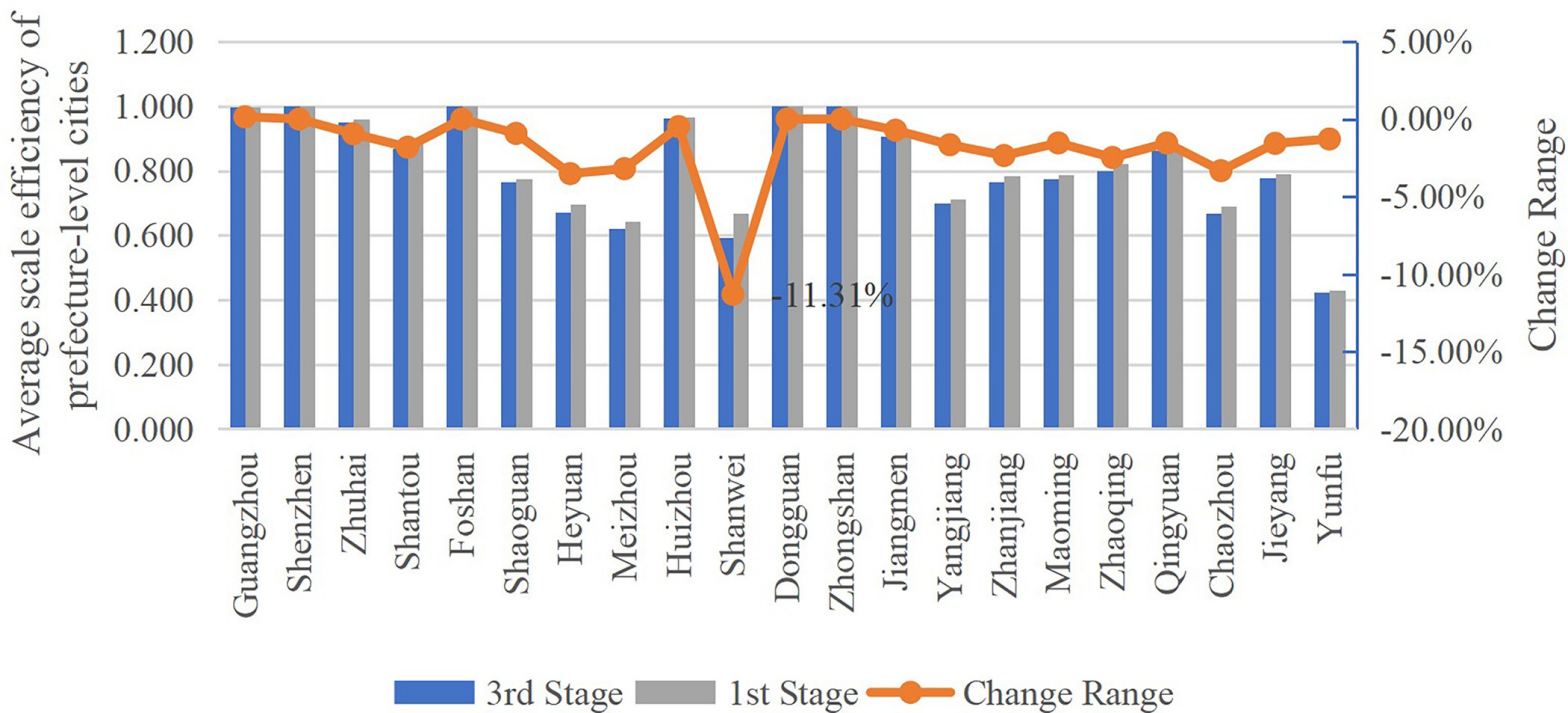
Change range of scale efficiency of the 3^rd^ stage compared to that of the 1^st^ stage.

As shown in Fig 3, from 2011 to 2020, the average scale efficiency of Stage 3 in the whole province decreased compared with that of Stage 1. Therefore, generally speaking, environmental factors have a positive impact on the scale efficiency of low-carbon economic development in Guangdong Province. Secondly, Shenzhen, Foshan, Dongguan and Zhongshan remain unchanged, and that of Guangzhou only increased 1.47% in 2018. After excluding environmental factors, the results are more representative. The development of low-carbon economy in the above five cities is relatively stable. The change range in scale efficiency of Shanwei in the 3^rd^ stage changed most than that in Stage 1 with the mean value of -11.31%, which shows that Shanwei has a good external environment. Thirdly, environmental factors have the most positive impact on East Guangdong area, followed by West Guangdong and North Guangdong, while they have little impact on Pearl River Delta. It proves that the low-carbon economy development in Pearl River Delta area is relatively stable.

### 5.4 Empirical analysis results of DEA-BCC model in the 3^rd^ stage

As mentioned above, environmental factors had some impacts on the efficiency of low carbon economic development in 21 prefecture-level cities of Guangdong Province. When excluding environmental factors, the results are more representative. The average efficiency values from 2011 to 2020 are divided into four levels from high to low (optimal -1, excellent -0.85–0.99, good -0.7–0.84, and relatively poor below 0.7). The results are shown in Table 8.

**Table 8:** Results of low-carbon economic development efficiency.

Grades	Cities
Best	Shenzhen, Foshan, Dongguan, Zhongshan
Excellent	Guangzhou, Zhuhai, Huizhou
Good	Shantou, Shaoguan, Jiangmen, Yangjiang, Maoming, Zhaoqing, Qingyuan, Jieyang
Relatively poor	Heyuan, Meizhou, Shanwei, Zhanjiang, Chaozhou, Yunfu

According to the three efficiency analysis results, the overall pure technical efficiency of Guangdong province is relatively high, with the average of 0.95. The comprehensive technical efficiency is reduced mostly because of the scale efficiency. Among 21 prefecture-level cities of Guangdong Province, Shenzhen, Foshan, Dongguan and Zhongshan are the most efficient in terms of efficiency of low-carbon economic development. All these cities are DEA effective with unchanged returns to scale. The current scale can be maintained. The development efficiency of Guangzhou, Zhuhai and Huizhou are excellent, at 0.99, 0.95 and 0.89, respectively. Shantou, Shaoguan, Jiangmen, Yangjiang, Maoming, Zhaoqing, Qingyuan and Jieyang have sound development efficiencies fluctuating between 0.7–0.84, while this number is relatively poor in Heyuan, Meizhou, Shanwei, Zhanjiang, Chaozhou and Yunfu with the comprehensive technical value lower than 0.7. Yunfu is the city with the lowest low-carbon economic development efficiency, only at 0.42, which proves that Yunfu is not very effective in low-carbon development. Moreover, the returns to scale of these cities increase in most years, that is, these cities can expand the existing scale of low-carbon economic development.

In addition, it is possible to improve technical efficiency by examining the level of input redundancy and output deficiency in cities whose pure technical efficiency is below the provincial average level and comparing the slack and surplus with the original input-output index. Table 9 displays the analysis findings. The redundancy of carbon dioxide emission is the highest, at 18.9%, followed by the redundancy of fixed assets stock and employment, at 4.4% and 4.2%, respectively. Redundancy of total energy consumption is the lowest at 0.5%. Therefore, controlling carbon dioxide emissions is the most effective way to improve the pure technical efficiency of low-carbon economic development, and it is also effective to reduce the redundancy of fixed assets and employment to some extent.

**Table 9:** Results of redundancy and insufficient output.

City	Pure Technical Efficiency	Redundancy of				Insufficient	
		Fixed Assets Stock (100 million RMB)	Employment Number (10,000 people)	Total Energy Consumption (10,000 tons of standard coal)	Carbon Dioxide Emission (Megatons)	GDP Output (100 million RMB)	Electricity Consump-tion of the Whole Society (KWH)
Shantou	0.849	407.3/4.9%	0/0%	0/0%	8.1/25.4%	240.5/9.2%	0/0%
Meizhou	0.840	0/0%	12.2/7.1%	0/0%	9.4/30.1%	5.4/0.4%	0/0%
Jiangmen	0.872	163.4/2.0%	0/0%	0/0%	5.2/19.2%	77.6/2.5%	0/0%
Zhanjiang	0.646	0/0%	6.5/1.6%	0/0%	14.7/24.5%	0/0%	0/0%
Maoming	0.867	0/0%	58.2/18%	36.9/3.4%	1.0/3.1%	0/0%	39.9/32.4%
Zhaoqing	0.814	271.0/4.0%	0/0%	0/0%	1.5/6.8%	80.5/3.7%	0/0%
Jieyang	0.865	1104/16.2%	0/0%	0/0%	0.5/2.4%	0/0%	0/0%
Average	0.822	278.0/4.4%	11.0/4.2%	5.3/0.5%	5.8/18.9%	57.7/2.3%	5.7/3.1%

### 5.5 Malmquist index analysis

The Malmquist index analysis on the data of the third stage can measure the dynamic change of the development efficiency of low-carbon economy. The results are shown in Table 10.

**Table 10:** Malmquist index analysis result.

Period	Total Factor Productivity TFP	Technical Efficiency Change effch	Technical Progress tech	Pure Technical Efficiency pech	Scale Efficiency sech
2011–2012	1.000	0.978	1.023	0.998	0.979
2012–2013	1.026	1.016	1.010	1.004	1.013
2013–2014	0.974	0.972	1.001	1.005	0.968
2014–2015	0.988	0.974	1.015	0.965	1.009
2015–2016	1.003	0.992	1.011	0.985	1.007
2016–2017	0.990	0.957	1.035	0.980	0.976
2017–2018	1.038	1.029	1.008	0.993	1.036
2018–2019	1.035	1.023	1.012	1.010	1.013
2019–2020	1.026	1.015	1.012	1.004	1.011
Average	1.009	0.995	1.014	0.994	1.001

As shown in Table 10, the TFP value of the third, fourth and sixth periods is less than 1, which shows that the development efficiency of low-carbon economy in Guangdong Province during these periods has decreased compared with the last period. The possible reason behind is the corresponding technological regression in different years or the reduction of scale efficiency. The TFP values of the second, fifth, seventh, eighth and ninth periods are greater than 1, indicating that the low-carbon economic development efficiency of Guangdong Province in these periods is increasing compared with the last period. Generally speaking, the development efficiency of low-carbon economy in Guangdong Province increased year by year from 2011 to 2013, fell to the lowest in 2014, and then fluctuated between 2015 and 2017. After 2017, the efficiency increases every year. However, the TFP value in the 7th period is greater than that in the 8th period and greater than that in the 9th period. It proves that the growth rate of low-carbon economic development efficiency in Guangdong Province slows down, which is mostly caused by the decline of scale efficiency.

## 6 Conclusions and suggestions

### 6.1 Conclusions

Based on the analysis on the development efficiency of low-carbon economy in Guangdong Province, the results are shown as follows:

(1) Shenzhen, Foshan, Dongguan, and Zhongshan were DEA effective in Stage 1 (2011 to 2020), with all three of their efficiency values being 1. With the exception of 2018, when Guangzhou’s scale efficiency had an impact and resulted in a tiny drop in its overall technical efficiency, all three of its efficiency ratings in Guangzhou were 1, making DEA effective. As a result, the top five cities have the highest rate of low-carbon economic development in the province.

(2) According to the results of Stage 3, the low-carbon economy development efficiency of 2 of 21 prefectural -level cities were excellent, 8 cities were good while 6 cities were relatively poor. 3 belongs to North Guangdong area, 2 belongs to East Guangdong area and 1 belongs to West Guangdong area. All the cities in the Pearl River Delta area have relatively high efficiency of low-carbon economic development.

(3) According to the analysis results of Malmquist index, the overall development efficiency of low-carbon economy in Guangdong Province was posing an increasing trend from 2011 to 2020. However, it slowed down gradually and declined in some years due to the impact of scale efficiency.

### 6.2 Suggestions

Firstly, it is advised to expand input in scale and improve scale efficiency. Except for Shenzhen, Foshan, Dongguan, and Zhongshan, all the other cities are typically in the incremental stage of returns to scale of low-carbon economic development. These cities can continue their existing scale of low-carbon economic development. Therefore, in order to stabilize the scale, the government may think about enlarging and modifying it. It is worth mentioning that the year 2018 is the only year that Guangzhou failed to achieve DEA efficiency and witnesses the decreased returns to scale. Therefore, Guangzhou needs to pay more attention to the scale efficiency of low-carbon economic development and maintain maximum output at a reasonable input scale.

Secondly, it is advised to make the most of environmental advantages and develop low-carbon economy. Environmental factors mostly have a positive impact on the low-carbon economy development efficiency in Guangdong Province, and has a great promoting effect on some cities, indicating that the current economy, market, policy, and other environmental factors are beneficiary to the development of low-carbon economy in Guangdong Province. Therefore, it is important for Guangdong Province to make the most of its environmental advantages, formulate low-carbon economic development policies suitable for local conditions. For example, it can create and enhance laws and regulations for combating climate change and reducing pollutant emissions, enhance the market price mechanism for trading carbon emissions, increase financial and tax support for the construction of green space infrastructure, and direct low-carbon investment and low-carbon funds to increase the transformation of technological innovation successes.

Thirdly, it is suggested to vigorously develop energy conservation and emission reduction technologies and adjust the industrial structure. For Guangdong Province, controlling carbon dioxide emissions is still the most effective way to improve technological efficiency. Optimizing the industrial, energy and transportation structure, increasing low-carbon infrastructure construction, supporting the development of green industries, improving energy utilization efficiency, and promoting the continuous reduction of carbon dioxide and air pollutant emissions will help gradually improve the recycling level of resources, and constantly develop and improve the green and low-carbon technology system. In the process of developing low-carbon economy, municipal governments should pay attention to carrying out emission reduction policies, developing green finance systems and promoting enterprise transformation in a multi-pronged way.

For future works, on the basis of the study, it is firstly suggested to investigate deeper in those low-carbon economy development relatively inefficient cities or areas. Especially, it is worthy of finding out methods to specifically eliminate the redundancy of those cities or areas. Secondly, since the growth rate of low-carbon economic development efficiency in Guangdong Province slows down and it is mostly caused by the decline of scale efficiency, it is recommended to further study how to maintain the scale efficiency. Last but not least, controlling carbon dioxide emissions is still the most effective way to improve technological efficiency, on such sense, future studies about how to control carbon dioxide emissions both from government and enterprises’ perspectives can be considered.
